# Variation in nitrogen partitioning and reproductive stage nitrogen remobilization determines nitrogen grain production efficiency (NUEg) in diverse rice genotypes under varying nitrogen supply

**DOI:** 10.3389/fpls.2023.1093581

**Published:** 2023-03-03

**Authors:** Birendra K. Padhan, Lekshmy Sathee, Santosh Kumar, Viswanathan Chinnusamy, Arvind Kumar

**Affiliations:** ^1^Division of Plant Physiology, ICAR-Indian Agricultural Research Institute, New Delhi, India; ^2^Division of Crop Research, Indian Council of Agricultural Research (ICAR) Research Complex for Eastern Region, Patna, Bihar, India; ^3^International Rice Research Institute (IRRI) South Asia Regional Centre (ISARC), Varanasi, Uttar Pradesh, India; ^4^International Crops Research Institute for the Semi-Arid Tropics, Patancheru, Telangana, India

**Keywords:** Nitrogen remobilization efficiency (NRE), Nitrogen Harvest Index (NHI), Nitrogen grain production efficiency (NUEg), Nitrogen use efficiency, Rice, NUE, Nitrogen deficiency, Optimum Nitrogen

## Abstract

Nitrogen (N) is an important macronutrient needed for grain yield, grain N and grain protein content in rice. Grain yield and quality are significantly determined by N availability. In this study, to understand the mechanisms associated with reproductive stage N remobilization and N partitioning to grain 2 years of field experiments were conducted with 30 diverse rice genotypes during 2019-Kharif and 2020-Kharif seasons. The experiments were conducted with two different N treatments; N deficient (N0-no external N application, available soil N; 2019-234.15 kgha-1, 2020-225.79 kgha-1) and N sufficient (N120-120 kgha-1 external N application, available soil N; 2019-363.77 kgha-1, 2020-367.95 kgha-1). N application increased the NDVI value, biomass accumulation, grain yield, harvest index and grain N accumulation. Post-anthesis N uptake and N remobilization from vegetative tissues to grain are critical for grain yield and N harvest index. Rice genotypes, Kalinga-1, BAM-4234, IR-8384-B-B102-3, Sahbhagi Dhan, BVD-109 and Nerica-L-42 showed a higher rate of N remobilization under N sufficient conditions. But, under N deficiency, rice genotypes-83929-B-B-291-3-1-1, BVD-109, IR-8384-B-B102-3 and BAM-4234 performed well showing higher N remobilization efficiency. The total amount of N remobilization was recorded to be high in the N120 treatment. The harvest index was higher in N120 during both the cropping seasons. RANBIR BASMATI, BAM-832, APO, BAM-247, IR-64, Vandana, and Nerica-L-44 were more efficient in N grain production efficiency under N deficient conditions. From this study, it is evident that higher grain N accumulation is not always associated with higher yield. IR-83929-B-B-291-3-1-1, Kalinga-1, APO, Pusa Basmati-1, and Nerica-L-44 performed well for different N use efficiency component traits under both N deficient (N0) and N sufficient (N120) conditions. Identifying genotypes/donors for N use efficiency-component traits is crucial in improving the fertilizer N recovery rate and site specific N management.

## Introduction

Rice (*Oryza sativa* L.) is an important food crop and a major dietary source of energy and proteins (Glangchai and Rangsri, 2021). Nitrogen (N) is an indispensable macronutrient for rice production and is responsible for protein accumulation in rice grains. N is directly associated with photosynthesis, biomass accumulation, tillering activity, spikelet formation, grain growth, and grain quality (Yoshida et al., 2006). N is one of the most critical yield-limiting factors in rice production and improving its utilization efficiency is essential for sustainable agriculture (Tilman et al., 2002; Hirel et al., 2007). However, crop intensification in the past with excessive N application and the decrease in the crop N recovery rate (<50%) has led to several environmental problems (Tilman et al., 2002). Indian soils are deficient in N (Dey and Sekhon, 2016) and more than 50% yield loss is common under N scarcity (Satyanarayana et al., 2012). Exogenous application of N fertilizer is mandatory to obtain higher yield returns from modern rice cultivars (Kraiser et al., 2011; Chamely et al., 2015). N fertilization at an adequate amount is critical for vegetative and reproductive growth in rice, and a better response to applied fertilizer is achieved in rice genotypes with higher yield potential (Rahman et al., 2007). However, excessive application of chemical fertilizers may have a negative environmental impact leading to pollution, biodiversity as well as yield loss (Cassman et al., 1998). It is estimated that ~ 60% of N inputs are in excess for major cereals including rice, and only 30%- 50% of applied N is taken up, and the unused N is lost to the environment (West et al., 2014; Herrera et al., 2016). Enhancing crop N use efficiency (NUE) will help in increasing yield under limited N conditions with minimum environmental impact (Moll et al., 1982). From a physiological point of view, NUE can be evaluated by the N utilization efficiency (NUtE) or N grain production efficiency (NUEg) (Ciampitti and Vyn, 2014). Of all the cereals, rice and wheat cropping systems have the lowest levels of nitrogen use efficiency (NUE), less than 30–40% (Norton et al., 2015; Taulemesse et al., 2015; Herrera et al., 2016). An additional amount of N fertilizer is applied to compensate for the effect of a lower uptake rate and to achieve higher grain yield (Elbasyoni et al., 2019). Reproductive stage N remobilization (NR) from the storage pool to sink organs is an important determinant for grain N recovery at harvest (Gaju et al., 2011). N assimilation associated with enhanced remobilization during seed filling leads to better yield, grain N accumulation, and protein content. N fertilizer management has a profound effect on grain yield and protein content (Zebarth et al., 2007). Improving the coordination between supply and crop demand, fertilizer management is a critical approach for simultaneously increasing grain yield and protein content (Chen et al., 2011). Better N management leads to higher absorption and a higher N harvest index (NHI) at crop maturity (Zheng et al., 2017). Experiments by Zhang et al. (2020) reported that adequate N supply increases post-anthesis N uptake and N remobilization to grain. The nutritional value of rice grain is largely determined by its protein content (Duan and Sun, 2005; Wenefrida et al., 2009). nutritional quality simultaneously in rice is a major challenge for researchers (Debata and Murty, 1986; Lim and Quah, 2007; Peng et al., 2007; Chen et al., 2012; Bhullar and Gruissem, 2013; Peng et al., 2014; Li et al., 2018).

Recycling of nutrients and metabolites takes place during grain filling along with the progression of leaf senescence. During the vegetative stage, young leaves are the major sink organs for N and metabolism, but during the reproductive stage, developing grain is the most important sink for the N pool. N is transported in the form of amino acids and serves as a precursor for the GS1-NADH-GOGAT reassimilation pathway in sink tissues. Most of the protein found in seeds is derived from the amino acids translocated after proteolysis from source organs. In rice and wheat, 80% of the total grain N is remobilized from leaves and other vegetative parts (Tabuchi et al., 2007; Xu et al., 2014). N in leaves is recycled following protein hydrolysis and exported to grains, 60% to 95% of grain N comes from the remobilization of N stored in shoots before anthesis. A less important fraction of seed N directly comes from post-flowering N uptake. The plant N status during the panicle initiation stage influences spikelet differentiation and yield. Before panicle emergence, most of the N is found in the leaves of rice. Following anthesis, there is a rapid transfer of leaf N to the developing panicles. Studies on the uptake and partitioning of N in rice showed that most of the N was distributed to new, actively growing organs. Deficiency in post-flowering N availability leads to a significant reduction in grain size and grain N content (DuPont and Altenbach, 2003). Zhang et al. (2020) found that the split application of N fertilizers increased N accumulation, which subsequently increased the protein content in grain. Proportionate allocation of N to component plant parts is important for higher efficiency of N translocation to the grain. Partitioning of N to vegetative plant parts during the vegetative stage is dependent on its availability in the soil, and its remobilization to the grain after anthesis is dependent on the N storage pool; the higher the storage of N in active vegetative organs the higher is the N remobilization activity (Nooden et al., 1997; Barraclough et al., 2014; He at al., 2016; He et al., 2016; Kumari et al., 2021).

Findings from various studies suggest that the NUE of rice cultivars varies in response to N fertilizer dose, which brings the opportunity and possibility to screen diverse rice genotypes for NUE component traits. We previously evaluated a set of 300 diverse rice genotypes in hydroponics and identified 30 genotypes with improved NUE (unpublished). The selected 30 genotypes were evaluated in the field for N utilization efficiency and variation in N assimilation (Jagadhesan et al., 2020; Jagadhesan et al., 2022). In this study, the selected 30 diverse genotypes were evaluated for NUE component traits in two consecutive seasons in the field. The aim was to understand the variations in vegetative greenness, biomass accumulation, harvest index, nitrogen and protein content, N remobilization efficiency, and N partition to different organs. The better-performing donor lines for different NUE component traits and surrogate traits are identified.

## Materials and methods

### Estimation of available soil nitrogen

In both the cropping seasons (2019-*Kharif* and 2020-*Kharif*) soil N availability was estimated following the Kjeldahl method (Bremner, 1960). The soil sample was collected from the field before cropping and was air-dried and soil N availability (kgha^-1^) was determined after digestion, distillation, and titration.

### Field experiments

Field experiments were conducted during the 2019-*Kharif* and 2020-*Kharif* seasons at the ICAR-Indian Agricultural Research Institute, New Delhi. A total of 30 diverse rice genotypes were selected to study reproductive stage nitrogen remobilization and associated traits with two different nitrogen application rates: N deficient (N0-without N application) and N sufficient (N120-N application at120 kgha^-1^). N was applied in split doses: 50% basal, 25% at active tillering, and 25% at booting. Phosphorous and potassium were applied as basal doses in both the treatments (N0 and N120) at 60 kg ha^-1^ P (SSP) and at 60 kg ha^-1^ K (MOP). In both seasons, soil N availability was estimated before and after crop growth.

### Estimation of biomass accumulation, partitioning, and yield-associated traits

Biomass accumulation was recorded from different plant parts: flag leaf, lower leaf, stem, and panicle after harvesting. Total biomass accumulation and biomass partitioning to individual part was recorded. The harvest Index was also calculated.

Harvest Index (HI)= (Panicle yield/Biological yield)*100

### Estimation of tissue nitrogen content

Tissue N content was estimated using the Kjeldahl method (Nelson and Sommers, 1980) from the samples collected during the 2019-*Kharif* season. Plant samples from different parts were collected separately (flag leaf, lower leaf, stem, and panicle) at two different stages: anthesis and maturity. Samples were oven dried to constant dry weight and made into powder to make the sample digestion easier. Protein content per plant was derived from the tissue N content using a conversion factor of 6.25 (Salo-vaananen and Koivistoinen, 1996; Mariotti et al., 2008).

### Estimation of nitrogen uptake and nitrogen grain production efficiency

To estimate the total N uptake, N content (mgg^-1^) was multiplied by total plant biomass. The result obtained was expressed as N uptake per plant. Nitrogen grain production efficiency was calculated as follows:

Nitrogen grain production efficiency (NUEg) = Panicle yield per plant/total nitrogen accumulation per plant

### Determination of N remobilization-associated traits

Different parameters associated with N movement within the plant parts were calculated (Cox et al., 1986). N Remobilization (NR) (mg plant^-1^) = N content at anthesis -N content at maturity; N Remobilization Efficiency (NRE) (%) = (N remobilization/N content at anthesis)*100; N lost or gained (kg ha^-1^) = N content at maturity - N content at anthesis; N at anthesis lost or gained (%) = (N lost or gained/N content at anthesis) × 100; N Harvest Index (NHI) = Grain N/total N content of aboveground parts at maturity; and N partitioning (%)= (N content in plant part/Total nitrogen content per plant)*100

### Statistical analysis

Two-way analysis of variance (ANOVA) of three biological replicates was carried out in GraphPad Prism version 8 (La Jolla, California, USA) with a variety of N treatments as treatment effects to compute adjusted P values and level of significance. Mean separation was done using Sidak’s multiple comparisons test following one-way ANOVA. Graphs and heat maps were prepared using GraphPad Prism version 8 (La Jolla, California, USA). A correlation study among different parameters was performed using a correlation plot package in R studio.

## Results

### Biomass accumulation, biomass partitioning, and Harvest Index

**Figure 1:**
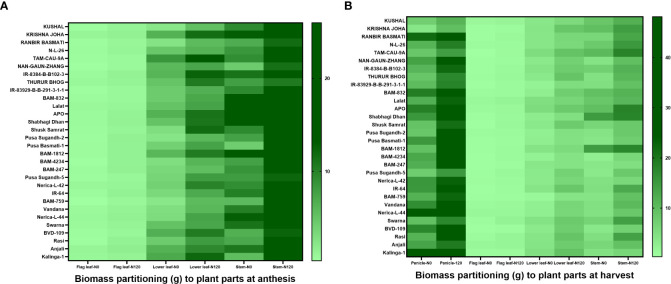
Effect of nitrogen deficient (0 kg ha-1 fertilizer N: N0) and nitrogen sufficient (120 kg ha-1 fertilizer N: N120) field conditions on biomass partitioning to plant parts at **(A)** anthesis (flag leaf, lower leaf, and stem) stage and **(B)** harvest (panicle, flag leaf, lower leaf, and stem) stage in field- grown diverse rice genotypes during the 2019-kharif season. Values presented are Mean±SE with three replications.

Soil nitrogen availability in both the experimental plots during the 2019 and 2020 seasons was in the low to medium range as shown in **Supplementary Table 1**. For 2019, N0-**234.15** kgha^-1^, and N120-**363.77** kgha^-1^ and for 2020, N0-**225.79** kgha^-1^, and N120-**367.95** kgha^-1^. Weather parameters were at optimum during the crop growing period (**Supplementary Figure 1**). Higher total biomass accumulation was recorded in both the cropping seasons in rice genotypes with N120 treatments in comparison to N0 (**Supplementary Figure 2**). During 2019, the highest biomass was recorded in APO (**72.94** g) in the N120 treatment and the lowest in Pusa Sugandh-5 (**14.65** g**)** in the N0 treatment. In 2020, the highest value of biomass accumulation was found in APO (**56.68** g) with the N120 treatment. But, the lowest value was found in KUSHAL (**15.76** g) with the N0 treatment. Total biomass accumulation among the genotypes was variable and was associated with Harvest Index and N remobilization activity. Biomass partitioning to different plant parts was also variable between treatments, stages, and genotypes. During the anthesis stage, the highest biomass partitioning to stem was recorded with the N120 treatment and lowest in the case of flag leaf in both treatments (**Figure 1A**). However, at harvest the result was different, and the highest biomass partitioning to panicle was recorded in both treatments (**Figure 1B**). A higher value was recorded in N120 in comparison to N0. Remobilization of stem reserve and photo-assimilate from leaves resulted in higher biomass partitioning to panicle resulting in higher yield. At the harvest stage, the lowest biomass partitioning was also found in the case of flag leaf under both treatments.

**Figure 2:**
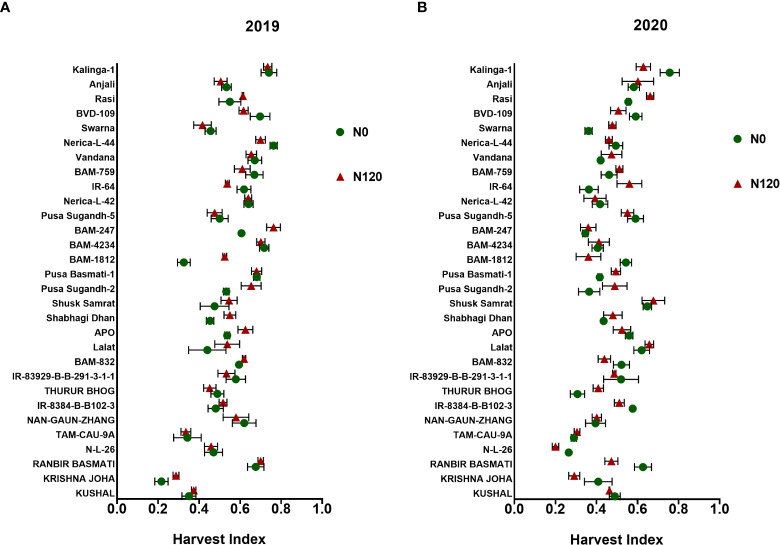
Effect of nitrogen deficient (0 kg ha-1 fertilizer N: N0) and nitrogen sufficient (120 kg ha-1 fertilizer N: N120) field conditions on Harvest Index during **(A)** the 2019-kharif and **(B)** the 2020-kharif seasons in field field-grown diverse rice genotypes. Values presented are Mean±SE with three replications.

N120 treatments maintained higher HI in comparison to N0 during both seasons (**Figure 2**). Though higher panicle yield was recorded in N120 treatments, HI was almost similar among the N treatments. Genotypes such as BAM-1812, Shabhagi Dhan, and APO accumulated higher vegetative biomass at harvest under N-deficient conditions. Higher HI values were obtained in the rice genotypes Nerica-L-44, Kalinga-1, and Shusk Samrat under the N0 condition. In terms of total biomass yield, the genotypes Kalinga-1, APO, and Shabhagi Dhan performed well in the N0 treatment.

### Nitrogen uptake, nitrogen remobilization

**Figure 3:**
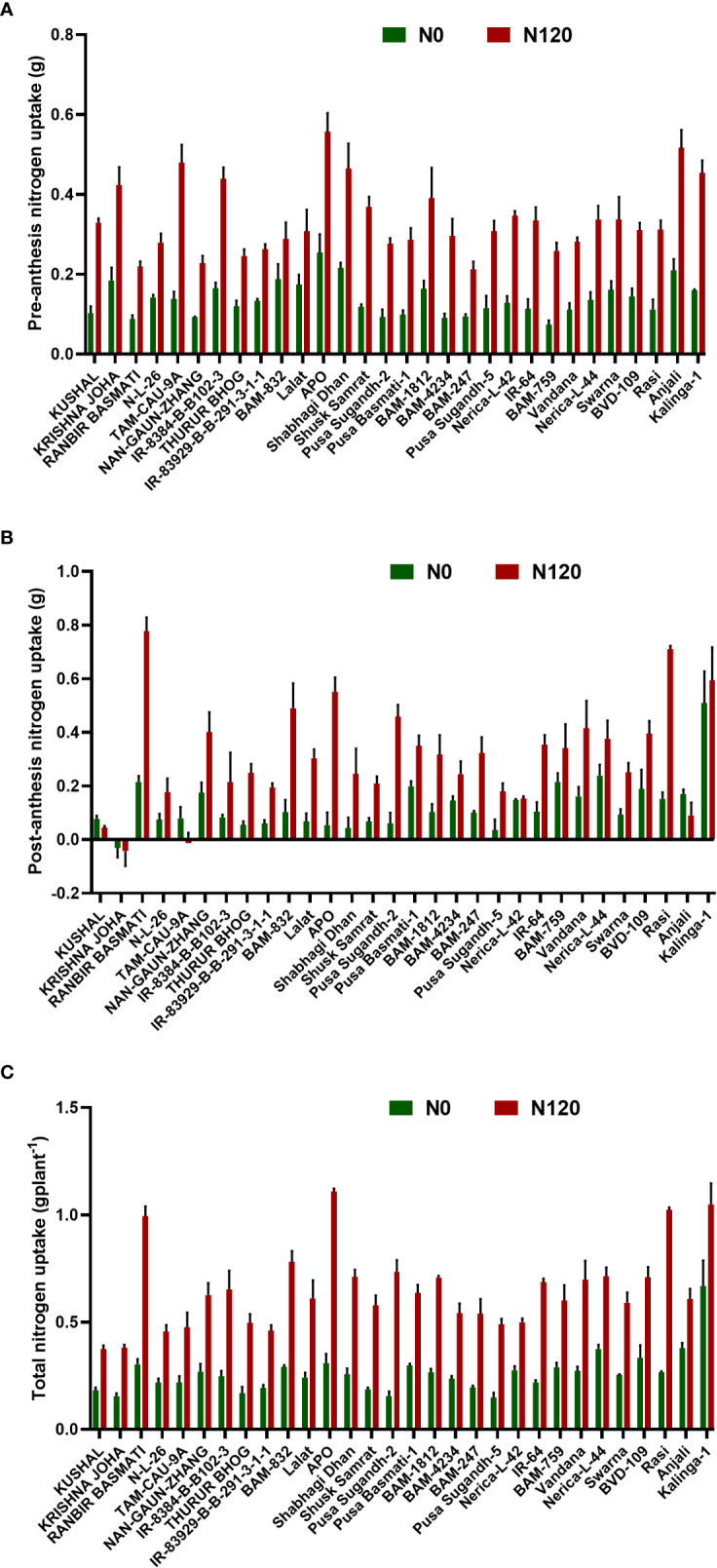
Effect of nitrogen deficient (0 kg ha-1 fertilizer N: N0) and nitrogen sufficient (120 kg ha^-1^ fertilizer N: N120) field conditions on **(A)** pre-anthesis nitrogen uptake and **(B)** post-anthesis nitrogen uptake, and **(C)** total nitrogen uptake in field- grown diverse rice genotypes during the 2019-kharif season. Values presented are Mean±SE with three replications.

**Figure 4:**
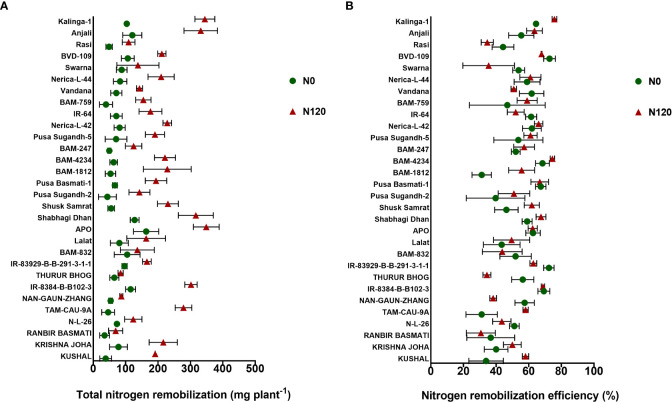
Effect of nitrogen deficient (0 kg ha-1 fertilizer N: N0) and nitrogen sufficient (120 kg ha-1 fertilizer N: N120) field conditions on **(A)** Total nitrogen remobilization and **(B)** Nitrogen remobilization efficiency in field-grown diverse rice genotypes during the 2019-kharif season. Values presented are Mean±SE with three replications.

Nitrogen uptake was higher in the N120 treatments in comparison to N0 at both pre-anthesis as well as post- anthesis stages (**Figures 3A, B**). Out of the total N taken up during the crop growth, a major part(~ 50-70%) was taken up before anthesis. Post-anthesis N uptake was 30-40% with few exceptions (RANBIR BASMATI, NAN-GAUN-ZHANG, BAM-832, Pusa Sugandh-2, BAM-4234, BAM-759, Nerica-L-44, and Rasi). The total N uptake at harvest was significantly higher in the N120 treatment than in the N0 treatment in all the genotypes (**Figure 3C**). RANBIR BASMATI, APO, Rasi, and Kalinga-1 displayed the highest N uptake among all the genotypes under N-sufficient conditions. The higher magnitude of post-anthesis N uptake contributed to the high total N uptake in these genotypes. A higher level of pre-anthesis uptake resulted in high N remobilization from vegetative organs to developing organs throughout the grain-filling stage. High N availability and storage in vegetative organs (flag leaf, lower leaf, and stem) resulted in higher N remobilization to developing grains. A significant difference in N remobilization activity was found between treatments in rice genotypes. Genotypes in the N120 treatment displayed higher total remobilization activity in comparison to N0 (**Figure 4A**). The highest values of N remobilization recorded were **348.79** mgplant^-1^**, 344.53** mgplant^-1^, and **331.84** mgplant^-1^ in APO, Kalinga-1, and Anjali, respectively (N120 treatment). The lowest values observed were **34.85** mgplant^-1^**, 38.22** mgplant^-1^, and **39.22** mgplant^-1^ in RANBIR BASMATI, KUSHAL, and BAM-759, respectively (N0 treatment). However, the N remobilization efficiency value was different from the total N remobilization (**Figure 4B**). Kalinga-1 **(75.76**%), BAM-4234 (**74.48%**), and IR-8384-B-B102-3 (**68.73%**) displayed the highest N remobilization efficiency under N-sufficient conditions. However, genotypes grown under N120 were more efficient in N remobilization. Total N remobilization and NRE were not always positively correlated across the genotypes studied. Some genotypes, such as IR-8384-B-B102-3, IR-83929-B-B-291-3-1-1, Pusa Basmati-1, BAM-4234, Nerica-L-42 and Vandana, with low total N remobilization under N deficient condition showed higher NRE. These results were more prevalent under the N0 condition. Some genotypes in the N0 treatment showed higher remobilization efficiency in comparison to the N120 treatment. The efficiency of N remobilization was between **31.15%** (N0) to **75.76%** (N120) across genotypes.

**Figure 5:**
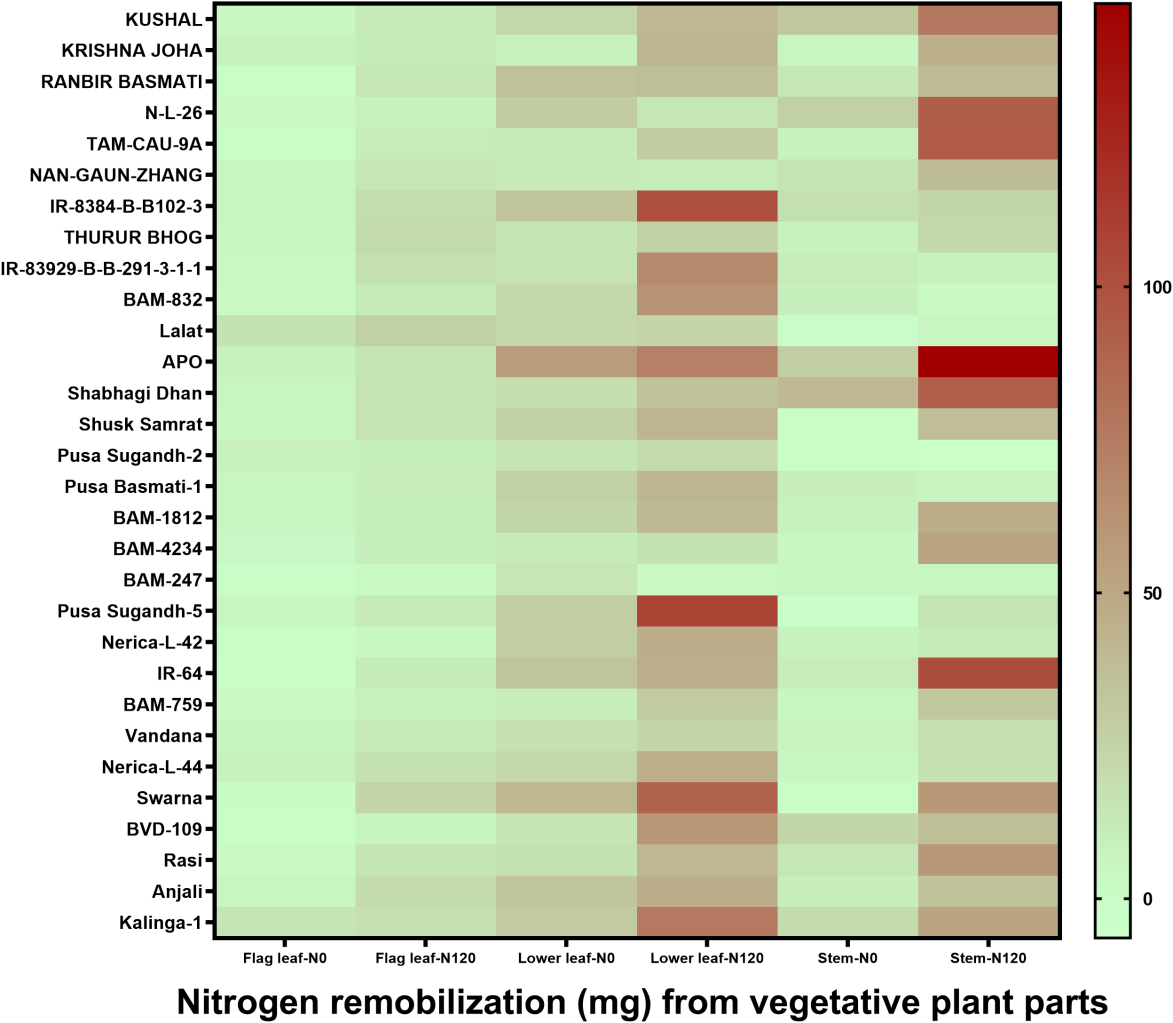
Effect of nitrogen deficient (0 kg ha-1 fertilizer N: N0) and nitrogen sufficient (120 kg ha-1 fertilizer N: N120) field conditions on nitrogen remobilization from vegetative plant parts (flag leaf, lower leaf, and stem) to grain in field- grown diverse rice genotypes during the 2019-kharif season. Values presented are Mean±SE with three replications.

N remobilization from vegetative plant parts, such as flag leaf, lower leaf, and stem, to grain was variable between treatments, between plant parts, and among genotypes (**Figure 5**). Among plant organs, the quantity of N remobilization was highest in the lower leaf followed by the stem and was at its lowest in the flag leaf. However, a higher value of N remobilization was observed among individual plant organs in the N120 treatment. Nitrogen remobilization percentage from the total N was highly variable (**Supplementary Figure 3**). The highest N remobilization percentage was observed in the flag leaf. Lower leaf and stem showed lesser N remobilization percentage from the total N content in the organs. In the case of the stem, a negative value in N remobilization was observed in some genotypes due to N losses from the plant system after anthesis. The total amount of N remobilization per plant stem contributed the highest percentage due to high stem biomass in comparison to flag leaf and lower leaf (**Supplementary Figure 4**). Flag leaf and lower leaf contributed a lesser percentage in N remobilization. However, the highest value was recorded in the flag leaf N remobilization percentage from the total N content in the flag leaf followed by the lower leaf and the lowest value was found in the stem.

Based on N remobilization efficiency, the rice genotypes can be classified into three groups: 1. High N remobilization (>60%), 2. Moderate N remobilization (40-60%), and 3. Low N remobilization (<40%). Genotypes such as IR-8384-B-B102-3, IR-83929-B-B-291-3-1-1, APO, Shabhagi Dhan, Shusk Samrat, Pusa Basmati-1, BAM-4234, Pusa Sugandh-5, Nerica-L-42, IR-64, Vandana, Nerica-L-44, BVD-109, Anjali, and Kalinga-1 fell under the high N remobilization category. Genotypes that fell under the medium N remobilization category were KUSHAL, KRISHNA JOHA, N-L-26, TAM-CAU-9A, THURUR BHOG, BAM-832, Lalat, Shabhagi Dhan, Shusk Samrat, Pusa Sugandh-2, BAM-1812, BAM-247, Pusa Sugandh-5, IR-64, BAM-759, Vandana, Nerica-L-44, Swarna, Rasi, and Anjali. RANBIR BASMATI was the only genotype that was included in the low N remobilization category. In terms of total N remobilization, the results were different. IR-8384-B-B102-3, APO, Shabhagi Dhan, Anjali, and Kalinga-1 displayed higher values in total N remobilization (> 300 mgplant^-1^) (**Figure 4A**). Concerning N uptake, genotypes such as APO and Kalinga-1 were more efficient under N-deficient conditions. NRE was found highest in IR-8384-B-B102-3, IR-83929-B-B-291-3-1-1, BAM-4234, and Pusa Basmati-1 with N0 treatment.

### Nitrogen Harvest Index, nitrogen partitioning, and total grain nitrogen accumulation

**Figure 6:**
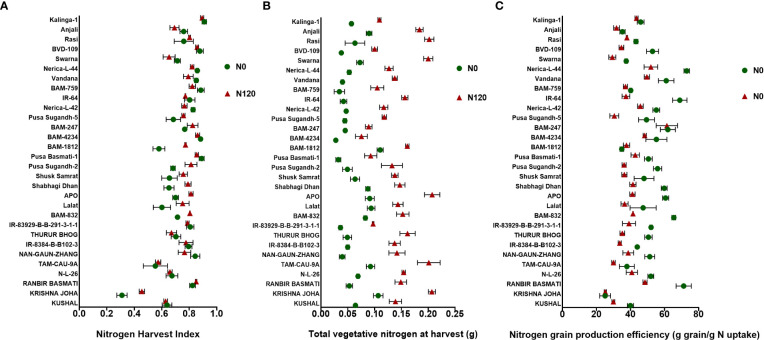
Effect of nitrogen deficient (0 kg ha-1 fertilizer N: N0) and nitrogen sufficient (120 kg ha-1 fertilizer N: N120) field conditions on **(A)** Nitrogen Harvest Index, **(B)** Total vegetative nitrogen at harvest, and **(C)** Nitrogen grain production efficiency in field- grown diverse rice genotypes during the 2019-kharif season. Values presented are Mean±SE with three replications.

**Figure 7:**
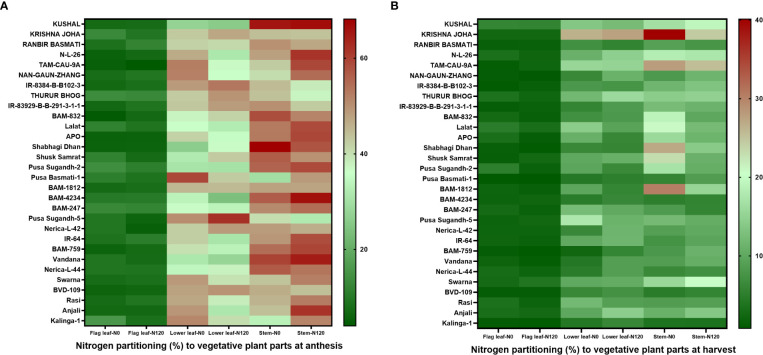
Effect of nitrogen deficient (0 kg ha-1 fertilizer N: N0) and nitrogen sufficient (120 kg ha-1 fertilizer N: N120) field conditions on nitrogen partitioning to vegetative plant parts (flag leaf, lower leaf, and stem) at **(A)** anthesis stage and at **(B)** harvest stage in field- grown diverse rice genotypes during the 2019-kharif season. Values presented are Mean±SE with three replications.

**Figure 8:**
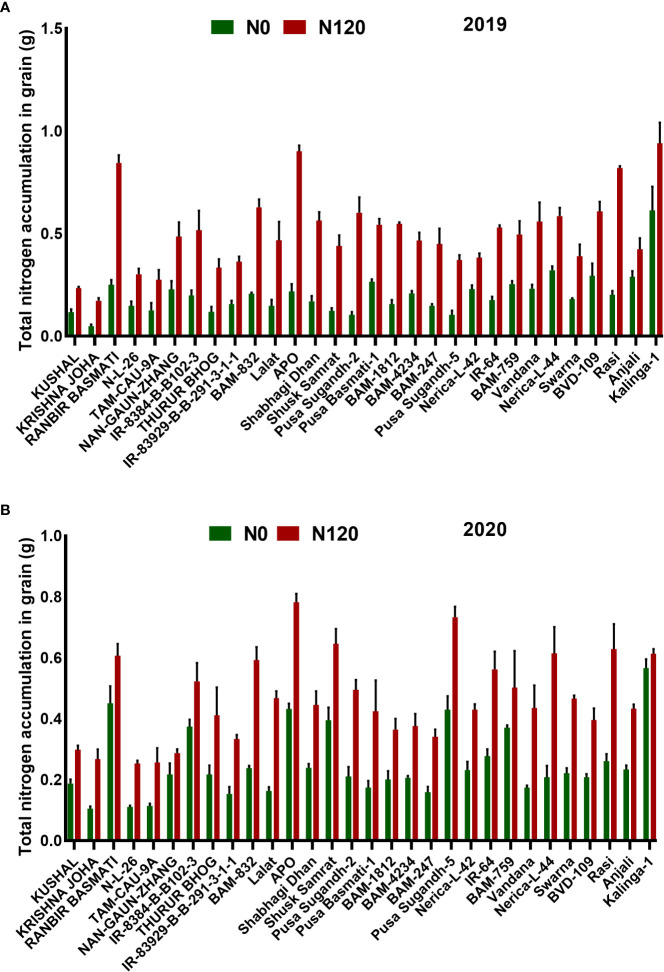
Effect of nitrogen deficient (0 kg ha-1 fertilizer N: N0) and nitrogen sufficient (120 kg ha-1 fertilizer N: N120) field conditions on total nitrogen accumulation in grain during **(A)** the 2019-kharif and during **(B)** the 2020-kharif cropping seasons in diverse rice genotypes. Values presented are Mean±SE with three replications.

**Figure 9:**
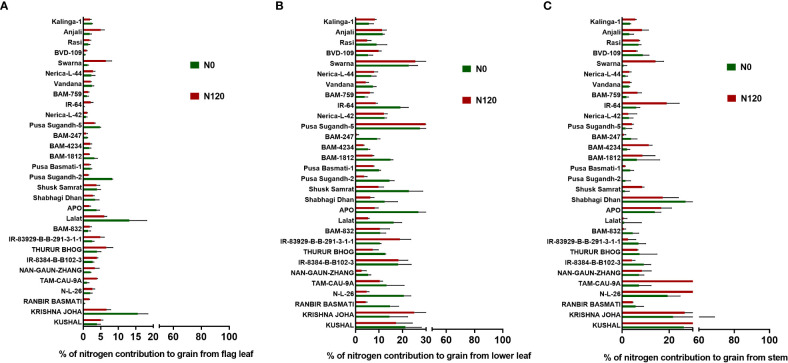
Effect of nitrogen deficient (0 kg ha-1 fertilizer N: N0) and nitrogen sufficient (120 kg ha-1 fertilizer N: N120) field conditions on **(A)** (%) percentage of nitrogen contribution to grain from flag leaf, **(B)**, (%) percentage of nitrogen contribution to grain from lower leaf, and **(C)** (%) percentage of nitrogen contribution to grain from the stem in field- grown diverse rice genotypes during the 2019-kharif season. Values presented are Mean±SE with three replications.

The NHI value ranges between **0.31** to **0.90** in the N0 treatment and between **0.45** to **0.89** in the N120 treatment among rice genotypes. Results were variable between treatments (N0 and N120) and among genotypes (**Figure 6A**). Higher NHI was reflected as lower N partitioning to vegetative parts and vice versa (**Figure 6B**). In the genotypes KRISHNA JOHA, TAM-CAU-9A, APO, Swarna, and Rasi total N accumulation in vegetative tissue was higher, but the NHI value was lower. Whereas the NHI value was higher in the genotypes Pusa Basmati-1, BAM-4234, BAM-759, BVD-109, and Kalinga-1. N content in vegetative tissue in these genotypes was lower. The NHI values were higher in the N0 treatment, whereas total vegetative N content was higher in the N120 treatment. Kalinga-1, Pusa Basmati-1, BAM-759, and BAM-4234 showed higher NHI values under the N0 condition. Variability in N partitioning to different vegetative plant parts, such as flag leaf, lower leaf, and stem, was observed. The total amount of N partitioned to stem was highest at the anthesis stage (**Figure 7A**) as well as at the harvest stage (**Figure 7B**). During the anthesis stage, higher N partitioning to vegetative parts was observed. During anthesis, the highest N partitioning was observed in the stem, followed by the lower leaf, and the lowest N partitioning value was observed in the flag leaf per total biomass of individual plant organs. The N partitioning per unit of biomass behaved in a reverse manner. The highest value was recorded for the flag leaf, followed by the lower leaf and the lowest N partitioning was observed in the stem. At maturity, the highest N partitioning was recorded from the panicle, represented as Nitrogen Harvest Index (**Figure 6A**). The NHI was positively correlated with total N accumulation in grain across genotypes. Higher NHI resulted in higher grain N accumulation. In both the cropping seasons, 2019 and 2020, total grain N accumulation was higher in the N120 treatment in comparison to the N0 treatment (**Figure 8**). During 2019, total grain N accumulation was between **0.10** g to **0.61** g in the N0 treatment and between **0.17** g to **0.93** g in the N120 treatment. During 2020, the value ranged between **0.10** g to **0.56 g** in the N0 treatment and between **0.2565 g** to **0.78 g** in the N120 treatment. During 2019, higher total grain N accumulation was recorded in comparison to 2020. In both seasons, Kalinga-1 showed the highest total grain N accumulation under N-deficient conditions. Percentage of N contributions from different vegetative parts to total grain N accumulation was found variable (**Figure 9**). Flag leaf contributed the lowest percentage of N to total grain N. Highest percentage of N was from the stem, followed by the lower leaf.

### Nitrogen grain production efficiency (NUEg), nitrogen loss or gain

**Figure 10:**
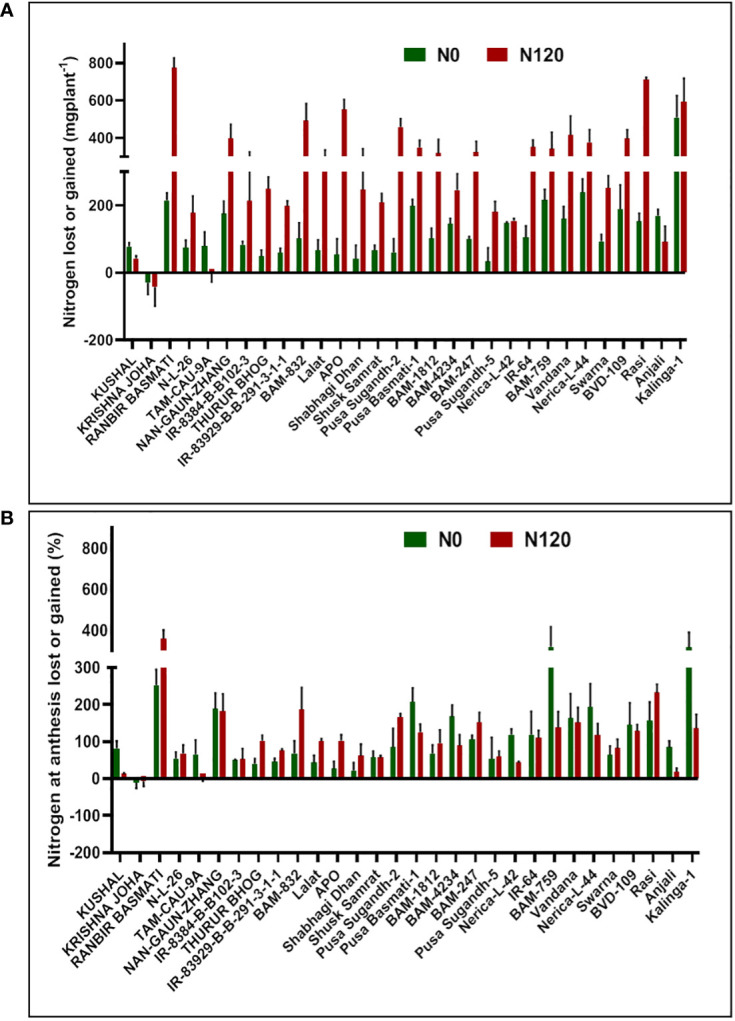
Effect of nitrogen deficient (0 kg ha^-1^ fertilizer N: N0) and nitrogen sufficient (120 kg ha^-1^ fertilizer N: N120) field conditions on **(A)** nitrogen lost or gained and **(B)** nitrogen at anthesis lost or gained in field-grown diverse rice genotypes during the 2019-*kharif* season. Values presented are Mean ± SE with three replications.

Nitrogen grain production efficiency (NUEg) was expressed as g grain produced per total N accumulation per plant. NUEg values were between **25.05** and **73.03** g grain/g N uptake (**Figure 6C**). Genotypes with the N0 treatment showed a higher value of NUEg in comparison to the N120 treatment. Genotypes without N application were more efficient in grain production with lesser N accumulation and grain produced per unit of available N was higher in comparison to genotypes with sufficient N application and higher tissue N content. However, the genotypes KRISHNA JOHA, BAM-1812, and BAM-247 performed well under N-sufficient conditions. Higher NUEg was observed with high N accumulation. Classifying all the genotypes studied into two groups, higher in NUEg and lower in NUEg (based on nitrogen grain production efficiency), the rice genotypes that fell under the higher NUEg group (>60 g grain/g N uptake) were RANBIR BASMATI, BAM-832, APO, BAM-247, IR-64, Vandana, and Nerica-L-44. The remaining genotypes could be placed in the lower NUEg group. The rice genotypes that displayed higher NUEg values under the N0 condition were RANBIR BASMATI, Nerica-L-44, IR-64, and BAM-832. In some genotypes, N absorbed during the vegetative stage was lost from the plant system after anthesis, resulting in lesser remobilization activity to developing grains during the reproductive stage. With few exceptions, in most of the genotypes, gain in N content was observed after anthesis (**Figure 10**). In KRISHNA JOHA (N0 and N120) and TAM-CAU-9A (N120), N loss was recorded. The gain in N content was predominant in N120 treated genotypes. However, the percentage gain in N was almost similar or also higher in the N0 treatments. The genotypes RANBIR BASMATI (N120), BAM-759 (N0), and Kalinga-1 (N0) showed exceptional percentage gain in N content after anthesis.

### Correlation among different NUE component traits

**Figure 11:**
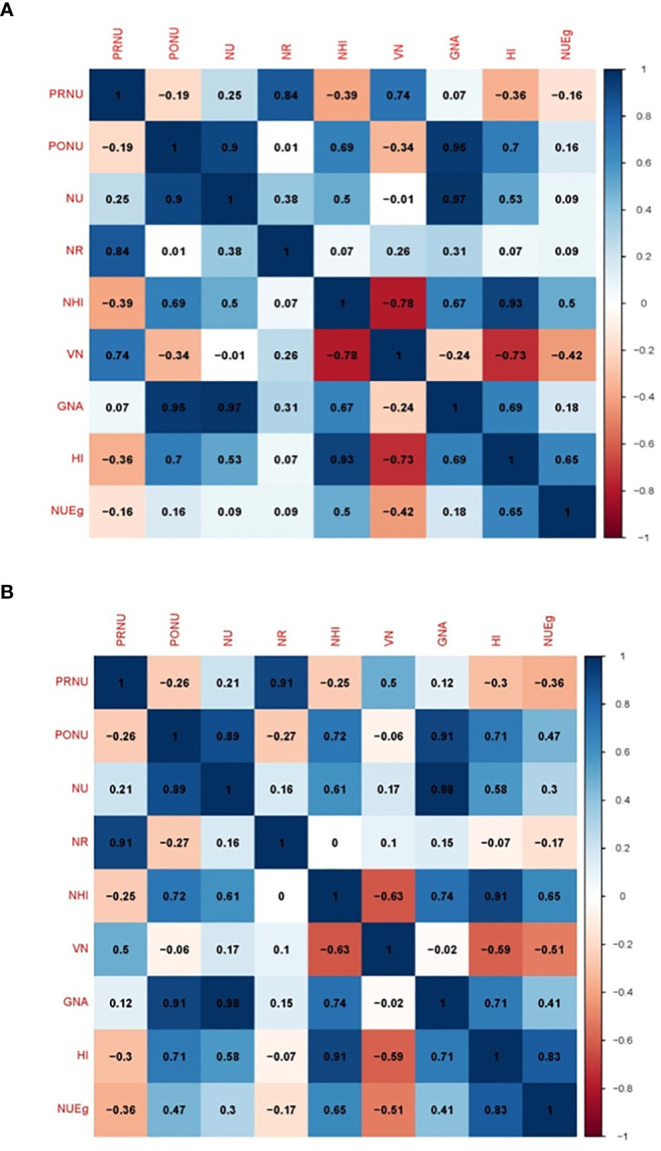
Pearson correlation matrix showing the relationship between PRNU (pre-anthesis nitrogen uptake), PONU (post-anthesis nitrogen uptake), NU (total nitrogen uptake), NR (nitrogen remobilization), NHI (nitrogen harvest index), VN (total vegetative nitrogen), GNA (total grain nitrogen accumulation), HI (harvest index), and NUEg (nitrogen grain production efficiency) under **(A)** nitrogen deficient (0 kg ha-1 fertilizer N: N0), **(B)** nitrogen sufficient (120 kg ha-1 fertilizer N: N120) field conditions.

Under the N deficient condition (N0), total N uptake was correlated to N remobilization, N Harvest Index, grain N accumulation, Harvest Index, and NUEg (**Figure 11A**). Under the N sufficient condition (N120), N uptake was positively correlated to all these parameters including total vegetative N. However, N uptake was strongly correlated to grain N accumulation under both N conditions. Pre-anthesis N uptake was strongly correlated to N remobilization under both conditions. However, post-anthesis N uptake showed a strong correlation with N remobilization and grain N accumulation under both N conditions. Pre-anthesis N uptake and post-anthesis N uptake showed negative correlations under both N conditions. With the N0 treatment, pre-anthesis N uptake showed a negative correlation with NHI, HI, and NUEg. Post-anthesis N uptake showed a negative correlation with total vegetative nitrogen (**Figure 11A**). With the N120 treatment, pre-anthesis N uptake showed a negative correlation with NHI, HI, and NUEg (**Figure 11B**). Post-anthesis N uptake showed a negative correlation with total vegetative N under the N0 treatment and with N remobilization and total vegetative N under the N120 treatment. NUEg was negatively correlated with pre-anthesis N uptake, N remobilization, and total vegetative N under the N120 condition (**Figure 11B**).

## Discussion

Nitrogen uptake, partitioning to different parts, and its remobilization during the reproductive stage towards the sink, i.e., developing grains, is important in improving N grain production efficiency, grain N, and grain protein accumulation (Good et al., 2004; Lin et al., 2006; Zhang et al., 2013; Hajibarat and Saidi, 2022; Khwankaew et al., 2022; Liu et al., 2022). Nitrogen recycling or remobilization from senescing plant parts and from the storage organs to sink tissues is accomplished after the onset of flowering and during the progress towards maturity (Lemaitre et al., 2008; Masclaux-Daubresse et al., 2008; Masclaux-Daubresse et al., 2010; He et al., 2016; Asibi et al., 2019; Melino et al., 2022). The major N storage organs include the stem, leaf, and petiole. The efficiency of remobilization is determined by the N uptake capacity and sink activity in developing grains (Martinoia et al., 1981; Lin et al., 2006). N remobilization or partitioning to grains and post-anthesis N uptake directly affects grain yield and quality (Ntanos and Koutroubas, 2002; Martin et al., 2006; Uauy et al., 2006; Masoni et al., 2007; Tabuchi et al., 2007; Xue and Yang, 2008; Li et al., 2013; Raddatz et al., 2020). Understanding the mechanisms controlling N remobilization during grain development and in response to stress and identifying the genotypes performing better under such environments is required to improve the N fertilizer economy.

### Nitrogen uptake improves biomass panicle yield, and Harvest Index

Nitrogen availability during vegetative growth is important for better reproductive stage development, yield, and a high Harvest Index. Higher N availability during the reproductive stage increases its partitioning to panicle as the developing grain acts as a sink increasing the demand for N from storage organs and remobilization activity (Dingkuhn, 1996; He et al., 2016; Kumari et al., 2021). Findings from previous studies showed that differential N application rates have a strong effect on source-sink activity in rice (Debata and Murty, 1986; Debata and Murty, 1988; Dingkuhn, 1996; Hameed et al., 2019; Chen et al., 2023). In our study, the soil N availability estimated in the experimental plots was between **200** kgha^-1^ to **400** kgha^-1^, which is not sufficient for optimum plant growth. An external source of N was supplied to study the effect on uptake, partitioning, remobilization, and grain growth of rice genotypes. The results showed a positive effect on biomass growth upon N application. N application promoted physiological activities and provided an optimum environment for plant growth. An increase in tiller number associated with plant growth in terms of height, leaf area, and leaf thickness culminated in high vegetative biomass. Few genotypes displayed better growth and biomass in low N, showing their efficiency and adaptability to the low N environment. Vegetative growth in the N0 treatment was supported by the soil N availabillity. Higher biomass accumulation accomplished due to high N availability and uptake resulted in high panicle yield and a higher Harvest Index value was obtained. Variability obtained in the HI was probably due to high vegetative N accumulation and lower level in partitioning to grain, which reduced the panicle biomass and HI. However, a higher HI was displayed with external N application supporting reproductive growth and better panicle yield. Withdrawal of N application to the field resulted in lower panicle yield and HI (Bahrani et al., 2011; Fageria, 2014; Qun et al., 2023).

### Post-anthesis nitrogen uptake and nitrogen remobilization were associated with Nitrogen Harvest Index, total nitrogen accumulation in grain, and panicle yield

Pre-anthesis N uptake is associated with remobilization and post-anthesis N uptake contributes to grain development and grain N and protein content. The N stored in the vegetative organs before anthesis plays an important role in maintaining high photosynthetic efficiency (Millard, 1988; Slafer and Satorre, 1999), but the higher N remobilization rate in the vegetative organs during grain filling may limit its accumulation (Rajcan and Tollenaar, 1999; Pommel et al., 2006). N movement takes place from source organs, mainly leaves, to sink organs, younger leaves during the vegetative stage and developing grains during the reproductive stage. Movement is dependent on the N status and developmental stage of the plant. When the N status is relatively low, plants remobilize N more efficiently. On the other hand, plants tend to retain N in source organs when N is sufficient (Assaf et al., 2014). This negative feedback regulation of N remobilization can act as a checkpoint to improve NRE in food crops. In our study, lower N during the vegetative stage was compensated by an uptake during the reproductive stage. Though the level of post-anthesis N uptake required for panicle yield and grain quality was not significant, it was very significant for total grain N accumulation. Post-anthesis N uptake and translocation are important for photosynthetic assimilation and grain filling in rice. Post-anthesis N uptake is critical as it is directly associated with N accumulation in rice grain. During reproductive development, the N absorbed by the plant is directly translocated to reproductive organs as the developing grain acts as a sink and the rate of N translocation is dependent on sink strength and activity. Higher N translocation to grain leads to higher total grain N accumulation and a high N Harvest Index (NHI). Improved coordination in N application and growth stages in rice can enhance N uptake while maintaining a high N harvest index (NHI) (Zheng et al., 2017). Therefore, some recent studies have shown that increasing the N uptake of crops after anthesis and its transport to the grain can increase protein content. Nitrogen partitioning during vegetative growth is critical for reproductive growth and panicle yield. Because the N content of different vegetative organs is critical for grain N content, N content in storage organs is translocated to the panicle and contributes to the growth of the panicle. The grain-filling period is critical for the formation of grain and identifying the physiological mechanisms of grain filling is desirable for higher yield (Ding et al., 2014). Findings in our study suggested that the NHI and total grain accumulation were positively correlated. Higher N partitioning to grain resulted in a higher NHI value and also higher panicle yield.However, higher N accumulation and NHI values were inversely correlated. Higher total N accumulation in vegetative tissues resulted in low N partitioning to grain. An increase in the N content of vegetative tissues decreased the N recovery amount and also the panicle yield. There is an inverse relationship between grain yield and total N accumulation in the plant (Cox et al., 1986; Clarke et al., 1990). So, crop genotypes that are efficient in panicle yield under N deficient or scarcity conditions are more desirable. Genotypes with such traits can be categorized under the high NUE group that is selected for crop breeding for resource use efficiency.

### Total nitrogen content and nitrogen loss determine nitrogen grain production efficiency

Nitrogen use efficiency is mainly determined by N uptake and its utilization by the plant. Any limitation to N uptake or utilization imposed by environmental, physiological, and agronomic processes is reflected in the form of yield loss and low NUE (Debaeke et al., 1996). Total grain yield or panicle yield per total available tissue N is variable. As more tissue N increased the NHI and grain protein content, the grain yield declined to show the opposite relationship between them. Higher grain yield or panicle yield resulted in dilution of N concentration in grain as well as in the plant. The grain N production efficiency (NUEg) was found higher with lower vegetative N accumulation and with the N0 treatment showing the inverse relationship between yield and total N accumulation. However, grain N accumulation had a positive relationship with yield. Higher grain N content was associated with higher grain growth activity and higher NUEg. The different hypotheses related to the inverse relationship between total N content and grain yield were: (i) dilution of N or protein in higher yield (Heitholt et al., 1990; Martre, 2003; Guarda et al., 2004; Barneix, 2007); (ii) higher rate of improvement in HI relative to NHI (Slafer et al., 1994); (iii) competition for energy and assimilates between biomass and N during the grain formation as some genotypes will continue to direct their energy and assimilate for vegetative growth after anthesis, and consequently less amount of assimilates for grain formation (Cox et al., 1985; Bogard et al., 2010); and (iv) difference in rate grain N and carbohydrates during the grain filling period (Jenner et al., 1991). Higher NUEg values were recorded in genotypes with no external N application as compared to the N120 application. However, grain N content was found higher in the N120 treatment. Other reasons for low NUEg are N loss or lower rate N gain at anthesis. The panicle yield will be lower if N present in the vegetative tissues is lost to the environment, which may happen through leaf fall or volatilization of secondary N compounds (Kanampiu et al., 1997; Wang et al., 2021). As N losses from the plant at anthesis were observed only in a few genotypes, the lower amount of N gain at anthesis may be the reason for low NUEg, though the grain yield and grain N content were high. As the N gain at anthesis through direct translocation to grain or remobilization from vegetative tissues promoted grain development, it had a positive relationship with yield. The lower value in gain of N anthesis also denotes the lower rate of N recovery; thus, N was lost from the vegetative biomass and lowering the in NUE.

Nitrogen Harvest Index and grain N concentration are essential for investigating changes in Nitrogen Use Efficiency (NUE) and grain quality attributes in rice (Heitholt et al., 1990). An increase in yield is usually reflected as a decline in grain N and protein concentration. As yield increment begins to stabilize at higher N uptake levels, grain N and protein concentration increases (McMullan et al., 1988; Barraclough et al., 2010; Sułek et al., 2023). Grain N concentration is usually source-limited (Borghi et al., 1986; Martre, 2003), particularly towards the end of the grain filling period, as opposed to grain yield being sink-limited (Jenner et al., 1991). When there is an N source limitation during grain filling, improving the coordination between soil N supply and crop N demand by N fertilizer management is a critical approach for simultaneously increasing the grain yield and protein content (Chen et al., 2011; Zhang et al., 2020; Qun et al., 2023). Therefore, in the case of no N application (N0 treatment), the source limitation was the major factor associated with higher N translocation to grain. In the N0 condition, the N uptake rate and the tissue N accumulation were lower. The N present in the vegetative tissues was remobilized efficiently to grain and displayed a higher NUEg. However, the gain in N content per plant in the N0 condition was lower.

**Figure 12:**
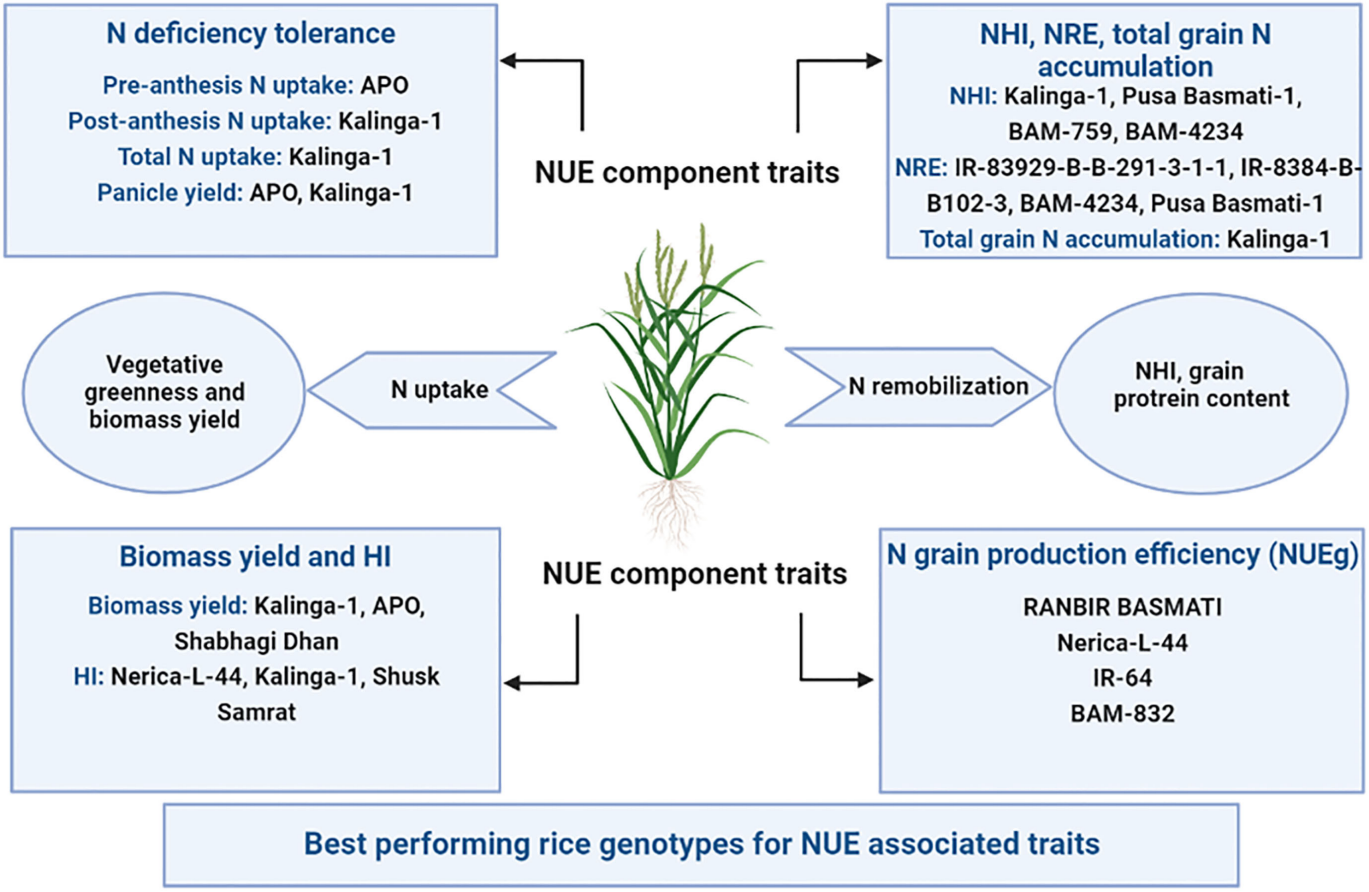
Schematic representation of N use efficiency (NUE) component traits in rice and best-performing genotypes under both nitrogen deficient (0 kg ha^-1^ fertilizer N: N0) and nitrogen sufficient (120 kg ha^-1^ fertilizer N: N120) field conditions. NHI, Nitrogen Harvest Index; NRE, Nitrogen Remobilization Efficiency; HI, Harvest Index; and NUEg, Nitrogen grain production efficiency.

IR-83929-B-B-291-3-1-1, Kalinga-1, APO, Pusa Basmati-1, and Nerica-L-44 were the rice genotypes that performed well for more than one N use efficiency component traits under both N deficient (N0) and N sufficient (N120) conditions (**Figure 12**). The N efficient rice genotypes had higher panicle yield and NUEg in comparison to inefficient genotypes under N scarcity. This is mainly due to larger sink size, higher sink strength, and improved filled grain percentage (Gallais and Hirel, 2004; Bahrani et al., 2011; Zhu et al., 2022; Qun et al., 2023). The genotypes performing better under N deficient conditions can be used as donors for N use efficiency component traits in breeding.

## Conclusion

Reproductive stage N remobilization is important for grain yield, grain N as well as protein content in rice. Higher uptake under sufficient N was associated with a high Normalized Difference Vegetation Index. N application promoted vegetative growth and more photosynthetic activity whereas an N deficient condition reduced panicle yield. Nitrogen application and its utilization increased the Harvest Index value and total N accumulation in plants. Soil N availability in the N0 plot was unable to support optimum plant growth and resulted in lower vegetative greenness and reduced yield. Though lower panicle yield was recorded under the N0 conditions, the nitrogen grain production efficiency was higher as most of the N present in the vegetative tissues were remobilized to grain and N recovery was high. More N accumulation under the N120 condition in vegetative tissues resulted in lower grain growth and yield. The N120 treatment enhanced pre-anthesis N uptake and more N remobilization to grain, resulting in high N remobilization efficiency. Post-anthesis N uptake and translocation to grain is an important determinant in improving grain yield as well as grain N content and higher Nitrogen Harvest Index. However, post-anthesis N uptake was lesser as compared to N uptake during the vegetative stage. So, the grain N content mainly relied upon vegetative N uptake and its remobilization during the reproductive stage to grain. N remobilization activity was found to be limited by source, hence lower N availability during the reproductive stage triggered more N translocation to developing grains. However, overall N remobilization, Nitrogen Harvest Index, and total N accumulation were higher in the case of the N120 treatment. Among various plant parts, the highest percentage of N remobilization from the total available N content was observed in the flag leaf, followed by the lower leaf, and the lowest percentage was observed in the stem. Unlike the percentage of N remobilization, total N remobilization was higher in quantity in lower leaf and stem as these parts contain higher total biomass. N loss from vegetative parts was also limited to N recovery and grain N accumulation. So, from these observations, it is concluded that genotypes under a low N environment with traits such as high N remobilization and partitioning, optimum yield, and higher NUEg are desirable for improving the N fertilizer economy and sustainable agriculture.

## Data availability statement

The original contributions presented in the study are included in the article/**Supplementary Material**. Further inquiries can be directed to the corresponding author.

## Author contributions

BK conducted experiments under the supervision of LS. BK performed the statistical analysis and wrote the manuscript. LS revised the manuscript. LS, VC, SK and AK finalized the experiments and manuscript. All authors contributed to the article and approved the submitted version.
