# Supplementary material for: Variation in nitrogen partitioning and reproductive stage nitrogen remobilization determines nitrogen grain production efficiency (NUEg) in diverse rice genotypes under varying nitrogen supply
Source: Front Plant Sci. 2023 Mar 3;14:1093581. doi: 10.3389/fpls.2023.1093581 (PMC10020356; doi:10.3389/fpls.2023.1093581)
Supplement: Supplementary file 1 [file DataSheet_1.docx]

**Supplementary table 1. Available soil nitrogen during 2019-*kharif* season and 2020-*kharif* season from the two different plots varying in nitrogen treatment (N deficient-N0, N sufficient-N120). Values presented are Mean ± SE with 3 replications.**

| Available soil nitrogen (kg ha^-1^) | | |
| --- | --- | --- |
| Cropping year | **N0** | **N120** |
| 2019 | 234.15 ±35.73 | 363.77±40.32 |
| 2020 | 225.79±19.16 | 367.95 ±22.13 |

**
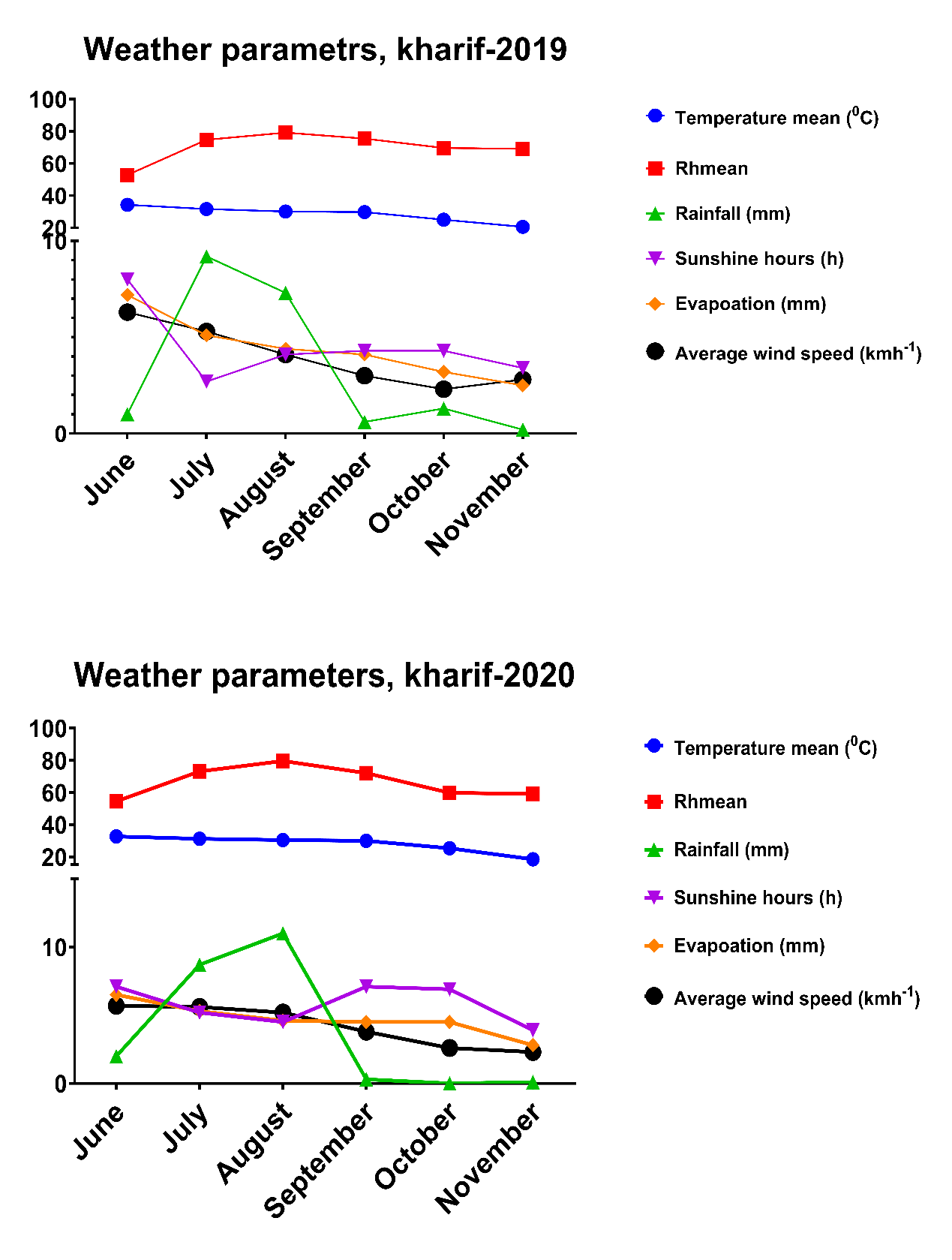
**

**(b)**

**(a)**

**Supplementary figure 1. Weather parameters recorded during (b) 2019-*kharif* season and (b) 2020-*kharif* season.**

**(a)**

**(b)**

**Supplementary figure 2. Total plant biomass accumulation at harvest during (a) 2019-*kharif* season and (b) 2020-*kharif* season in diverse rice genotypes under two different nitrogen treatments (N deficient-N0, N sufficient-N120). Values presented are Mean ± SE with 3 replications.**

**(a)**

**(b)**

**(c)**

**Supplementary figure 3. Nitrogen remobilization % from total (a) flag leaf nitrogen content, (b) lower leaf nitrogen content, and (c) stem nitrogen content during 2019-*kharif* season in diverse rice genotypes under two different nitrogen treatments (N deficient-N0, N sufficient-N120). Values presented are Mean ± SE with 3 replications.**

**(b)**

**(a)**

**(c)**

**Supplementary figure 4. Nitrogen remobilization % from (a) flag leaf nitrogen, (b) lower leaf nitrogen, and (c) stem with respect to total nitrogen remobilization per plant during 2019-*kharif*  season in diverse rice genotypes under two different nitrogen treatments (N deficient-N0, N sufficient-N120). Values presented are Mean ± SE with 3 replications.**
